# Prenatal alcohol exposure dysregulates spinal and circulating immune cell circular RNA expression in adult female rats with chronic sciatic neuropathy

**DOI:** 10.3389/fnins.2023.1180308

**Published:** 2023-06-09

**Authors:** Shahani Noor, Ariana N. Pritha, Andrea A. Pasmay, Jacob E. Sanchez, Joshua J. Sanchez, Annette K. Fernandez-Oropeza, Melody S. Sun, Michela Dell’Orco, Suzy Davies, Daniel D. Savage, Nikolaos Mellios, Erin D. Milligan

**Affiliations:** Department of Neurosciences, School of Medicine, University of New Mexico, Albuquerque, NM, United States

**Keywords:** prenatal alcohol exposure, neuroimmune, circular RNA, nerve injury, biomarker, NF-kB, blood leukocytes, TLR4

## Abstract

Alcohol consumption during pregnancy is associated with Fetal Alcohol Spectrum Disorders (FASD) that results in a continuum of central nervous system (CNS) deficits. Emerging evidence from both preclinical and clinical studies indicate that the biological vulnerability to chronic CNS disease in FASD populations is driven by aberrant neuroimmune actions. Our prior studies suggest that, following minor nerve injury, prenatal alcohol exposure (PAE) is a risk factor for developing adult-onset chronic pathological touch sensitivity or allodynia. Allodynia in PAE rats occurs concurrently with heightened proinflammatory peripheral and spinal glial-immune activation. However, minor nerve-injured control rats remain non-allodynic, and corresponding proinflammatory factors are unaltered. A comprehensive molecular understanding of the mechanism(s) that underlie PAE-induced proinflammatory bias during adulthood remains elusive. Non-coding circular RNAs (circRNAs) are emerging as novel modulators of gene expression. Here, we hypothesized that PAE induces dysregulation of circRNAs that are linked to immune function under basal and nerve-injured conditions during adulthood. Utilizing a microarray platform, we carried out the first systematic profiling of circRNAs in adult PAE rats, prior to and after minor nerve injury. The results demonstrate a unique circRNA profile in adult PAE rats without injury; 18 circRNAs in blood and 32 spinal circRNAs were differentially regulated. Following minor nerve injury, more than 100 differentially regulated spinal circRNAs were observed in allodynic PAE rats. Bioinformatic analysis identified that the parental genes of these circRNAs are linked to the NF-κB complex, a central transcription factor for pain-relevant proinflammatory cytokines. Quantitative real-time PCR was employed to measure levels of selected circRNAs and linear mRNA isoforms. We have validated that *circVopp1* was significantly downregulated in blood leukocytes in PAE rats, concurrent with downregulation of *Vopp1* mRNA levels. Spinal *circVopp1* levels were upregulated in PAE rats, regardless of nerve injury. Additionally, PAE downregulated levels of *circItch* and *circRps6ka3*, which are linked to immune regulation. These results demonstrate that PAE exerts long-lasting dysregulation of circRNA expression in blood leukocytes and the spinal cord. Moreover, the spinal circRNA expression profile following peripheral nerve injury is differentially modulated by PAE, potentially contributing to PAE-induced neuroimmune dysregulation.

## 1. Introduction

Exposure to alcohol during gestation can cause mild to severe cognitive and behavioral deficits, representing a continuum referred to as Fetal Alcohol Spectrum Disorders (FASD). While FASD occurs worldwide, the prevalence in the US is as high as 5% in some regions (England et al., 2020), indicating it is a significant public health problem with increasing medical costs to support the health and well-being of these populations. Prenatal alcohol exposure (PAE), which leads to FASD, confers well-characterized long-term physical health problems that impact central nervous system (CNS) function (Panczakiewicz et al., 2016; Reid et al., 2021). Altered immune system function in individuals with FASD (Tominaga and Caterina, 2004; Topiwala et al., 2017) has been implicated in chronic disease, as evidenced by a 4.9-fold greater incidence of autoimmune disorders (Himmelrich et al., 2000). Evidence of immune dysfunction as a consequence of PAE is also linked with Type II diabetes, allergy, and asthma in clinical studies (Zhang et al., 2005; Salmon, 2018). Several preclinical studies reported heightened brain proinflammatory cytokines due to PAE (Drew et al., 2015; Terasaki and Schwarz, 2016; Pascual et al., 2017). Both animal and clinical studies show that the underlying biological vulnerability due to PAE that extends to chronic CNS disease is driven by dysfunctional CNS-immune interactions (Drew and Kane, 2014; Wilhelm and Guizzetti, 2015; Terasaki and Schwarz, 2017; Bodnar et al., 2018, 2020; Ruyak et al., 2022). Little is known about how PAE-related changes lead to chronic CNS dysfunction triggered by challenges in adulthood.

Sensory processing disorders such as touch hypersensitivity are one of the comorbid conditions observed in children with FASD (Franklin et al., 2008; Moore and Riley, 2015; Fjeldsted and Xue, 2019). Notably, hypersensitivity to light touch referred to as mechanical allodynia, is a manifestation of peripheral neuropathy from chronic CNS dysfunction (Milligan and Watkins, 2009). Our recent reports demonstrate that moderate PAE is a risk factor for developing allodynia, following minor, peripheral sciatic nerve chronic constriction injury (CCI) in adult rats (Sanchez et al., 2017; Noor et al., 2020). Strikingly, a PAE-generated heightened susceptibility to develop allodynia is unmasked *only after* a minor nerve injury. Allodynia in PAE offspring occurs concurrently with augmented production of well-defined pain-relevant proinflammatory immune signaling in the spinal cord (Noor et al., 2017, 2020; Sanchez et al., 2017), where damaged peripheral axon terminals communicate with spinal pain neurons. Moreover, inhibiting peripheral and spinal proinflammatory immune activation is sufficient to reverse chronic allodynia in nerve-injured PAE rats (Sanchez et al., 2019; Noor et al., 2020). Similarly, our prior report and others described a PAE-induced proinflammatory bias with increased levels of Interleukin (IL)-1β and tumor necrosis factor (TNF)-α production from peripheral leukocytes upon immune stimulation with lipopolysaccharide (LPS) (Terasaki and Schwarz, 2016; Sanchez et al., 2017). These data are suggestive of PAE-induced long-term immune dysregulation. However, the overt basal identification of key peripheral and central immune modulators as a consequence of PAE is yet to be determined (Noor and Milligan, 2018).

The mechanism of long-term modulatory effects of PAE on neuroimmune interactions during adulthood remains poorly understood (Sanchez et al., 2019; Noor et al., 2020). Emerging evidence suggests that epigenetic mechanisms involving non-coding RNAs are strong mediators of long-lasting effects of PAE (Savage et al., 2010; Balaraman et al., 2013, 2014, 2016; Tseng et al., 2019; Mahnke et al., 2021). Although several studies have focused on the roles of non-coding RNAs in neuronal function, their roles within the context of PAE-induced neuroinflammatory consequences are not known. Circular RNAs (circRNAs) are a novel category of long non-coding RNAs that are derived from the circularization and covalent joining of back-spliced exons and/or introns (Barrett and Salzman, 2016; Haque and Harries, 2017), where the 3′ terminus of a downstream exon ligates to the 5′ terminus of an upstream exon, compared to linear mRNA transcripts generated from canonical splicing. Recent advances in high-throughput RNA sequencing and bioinformatic analysis approaches have revealed the existence of tens of thousands circRNAs in multiple species (Barrett and Salzman, 2016; Haque and Harries, 2017; Ng et al., 2018). CircRNAs exhibit high abundance, stability, tissue specificity and expression during discrete developmental stages of the CNS (Peng Peng et al., 2021). More recently, Mellios et al., reported evidence of circRNA alterations in developing and postnatal brains in a mouse model of PAE (Paudel et al., 2020; Papageorgiou et al., 2023). In the current set of studies, we hypothesized that PAE induces dysregulation of circRNAs under basal and nerve-injured conditions that are associated with immune function during adulthood. Utilizing a microarray-based technology, we evaluated levels of circRNAs in the spinal cord in adult non-PAE and PAE rats with or without minor nerve injury. Additionally, we investigated whether PAE-induced dysregulation of circRNAs was detectable in circulating immune cells from the blood in adult rats. A comprehensive bioinformatic analysis and additional validation studies of selected circRNAs, along with mRNA levels of these parental or host genes (i.e., genes giving rise to a specific circRNA), was carried out to explore circRNAs concurrent with PAE-related neuropathic pain susceptibility in adult rats.

## 2. Materials and methods

### 2.1. Animals

All procedures were approved by the Institutional Animal Care and Use Committee (IACUC) of The University of New Mexico Health Sciences Center. All animal experiments were carried out in accordance with ARRIVE (Animal Research: Reporting of *In Vivo* Experiments) guidelines and National Institutes of Health guide for the care and use of laboratory animals (NIH Publications No. 8023, revised 1978). Long-Evans rat breeders were purchased from Harlan Industries (Indianapolis, IN) and were maintained in a breeding colony on a 12:12-h reverse light/dark schedule (lights on from 2,100 to 0900 h), and fed Teklad 2,920X rodent chow and tap water, available *ad libitum*. For all experiments, 6- to 7-month-old female prenatal alcohol-exposed (PAE) or age-matched Saccharin control (Sac control) rat offspring were used. Offspring were habituated to a standard light/dark cycle (lights on from 0600 h to 1800 h) for at least 28 days and kept in these conditions for the duration of the study.

### 2.2. Moderate prenatal alcohol exposure paradigm

Pregnant female rat dams were given either ethanol or saccharin throughout pregnancy until birth according to the well-established drinking paradigm described in prior reports (Hamilton et al., 2010; Noor et al., 2017; Sanchez et al., 2017, 2019; Davies et al., 2019). Briefly, after 1 week of acclimation to the animal facility, all 3 month-old female breeders were single housed and allowed to drink tap water containing 0.066% (w/v) Saccharin that gradually increased in ethanol content from 0% (v/v) on Days 1–2, to 2.5% (v/v) on Days 3–4, to 5% (v/v) on Day 5 and thereafter for 2 weeks. Females were allowed to drink for 4 h each day from 1,000 to 1,400 h. Regular drinking water was freely available from 1,400 h to 1,000 h the next morning. Daily, 4-h ethanol consumption was monitored for 2 weeks after which the mean daily ethanol consumption was determined for each female. Females, whose mean daily ethanol consumption was greater than one standard deviation below the group mean, typically about 10–15% of the entire group, were removed from the study at this point. Subsequently, the remainder of the females were assigned to either a Saccharin control (Sac) or 5% ethanol drinking group and matched such that the mean pre-pregnancy ethanol consumption by each group was similar. Subsequently, females were placed with proven male rat breeders until pregnant. No alcohol was consumed during the breeding period, which averaged between 1 and 2 days.

Beginning on gestational day 1 (GD1), rat dams were given either 0% (Sac) or 5% ethanol (PAE) in Sac water (4 h/day). Sac control group rats were given a volume of 0% ethanol in Sac water that was matched to the mean volume consumed by the PAE group. The amount of ethanol consumed was determined 4 h after the drinking tubes were introduced into the cages for each dam through GD21. Ethanol consumption was discontinued at birth. Offspring were weaned at 24 days of age and offspring were pair-housed. Serum ethanol concentrations were measured from a separate set of rat dams. Blood samples were collected from tail veins and trunk blood, and average values from these samples are reported.

Our prior study unmasked unique signatures of immune-related factors underlying the risk of neuropathy from PAE in female rodent offspring. Critical contributions of peripheral immune cells were identified underlying spinal glial-immune proinflammatory activation (Noor et al., 2020). To further characterize the underlying factors leading to neuroimmune activation during nerve injury, we have carried out these studies in females. The potential effect of different phases of the estrous cycle on hindpaw threshold responses was not systematically examined. However, despite that all female offspring were housed in the same colony room, characterization of hindpaw response thresholds demonstrated <10% variance at baseline and after surgical manipulation. These observations were consistent despite female rats entering experiments at different phases of the estrous cycle, suggesting that hindpaw responses remained independent of the estrus cycle phase. Consequently, the stage of the estrous cycle was not a controlled variable, and female rats were randomly entered into and examined throughout the chronic neuropathy paradigm. It should be noted that none of the experimental groups contained more than one subject from a given litter in order to avoid “litter effects.”

### 2.3. Minor chronic constriction injury

Chronic constriction injury (CCI) is a well-established model of mechanical allodynia (Bennett and Xie, 1988) involving the application of 4 segments of 4-0 chromic gut sutures around the sciatic nerve. The material of the chromic gut induces peri-neural inflammation, causing edema and slight compression of the nerve. This model of experimental neuropathy is an ideal model to reflect peripheral neuropathy, as about 50% of all peripheral neuropathies in humans involve both direct nerve damage and peri-neural inflammation. In contrast to other models of peripheral neuropathy that cause a complete disruption of the peripheral nerves (Menorca et al., 2013; Challa, 2015), the sciatic nerve CCI model allows a systematic reduction of the degree of sciatic nerve damage. In our prior reports, we modified the standard CCI model to produce a minor CCI injury that applies a *single suture,* rather than 4 suture segments, to minimize the magnitude of nerve injury (Sanchez et al., 2017, 2019; Noor et al., 2020). This modification was developed to unmask the greater risk of PAE offspring developing neuropathy, compared to Sac-exposed control rats. All surgical procedures were performed under aseptic techniques as previously described (Noor et al., 2017, 2020; Sanchez et al., 2017, 2019). Briefly, under isoflurane anesthesia, the sciatic nerve was carefully isolated and loosely ligated with 1 segment of 4–0 chromic gut suture (Ethicon, Somerville, NJ) without pinching into the nerve. Sham surgery involved isolation of the sciatic nerve, without nerve ligation. The overlying muscle was sutured closed with two 3-0 sterile silk sutures (Ethicon, Somerville, NJ). All rats fully recovered from anesthesia within approximately 5 min. Animals were monitored daily after surgery.

### 2.4. Behavioral assessment of allodynia

Hindpaw touch threshold was measured utilizing the von Frey fiber test method, as described in our prior reports (Milligan et al., 2000; Sanchez et al., 2017, 2019; Noor et al., 2020). Briefly, rats were habituated to the testing environment atop 2-mm thick parallel bars spaced 8-mm apart allowing full access to the plantar hindpaw. Habituation occurred for approximately 45 min per day for 4 sequential days within the first 3 h of the light cycle (inactive phase) in a sound- and temperature-controlled dimly lit section of the home colony room. Baseline (BL) responses were then assessed. A total of 13 calibrated monofilaments (touch-test sensory evaluator, North Coast Medical, United States) were used in these studies, with the range from 3.61 to 5.18 log stimulus intensity. Following our previously published protocol (Noor et al., 2017; Sanchez et al., 2017, 2019), fibers were applied to the plantar surface of the left and right hindpaw for a maximum of 8 s per application. Random order between left and right hindpaw assessment was conducted. A metronome placed in the room provided guidance at 1 tick/s. Lifting, licking, or shaking the paw was considered a response. In a similar manner to BL evaluation, all rats were re-assessed following CCI or Sham surgery on Days 3, 10, 17, 24, and 28. The experimental testers were blinded to the treatment groups.

### 2.5. Blood leukocyte and spinal cord tissue collection and RNA extraction

Immediately following behavioral analysis on Day 28 after CCI surgery, rats were deeply anesthetized under isoflurane, 8–10 min, 5% vol in oxygen. 1 mL blood sample was collected through cardiac puncture using a 25G^5/8^ needle, and a 3 mL syringe (Becton Dickinson, United States). Blood samples were immediately processed to isolate blood circulating peripheral leukocytes (peripheral blood mononuclear cells) utilizing Ficoll density gradient centrifugation (GE Healthcare, IL, United States), as described in our prior report (Sanchez et al., 2017). Following blood collection, rats went through rapid transcardiac perfusion with ice-cold 0.1 M phosphate-buffered saline (PBS; pH = 7.4; flow rate 10 mL/min). Rats were placed on a frozen gel refrigerant pack (Glacier Ice, Pelton Shepherd Industries) and the lumbar spinal cord (LSC; L3-L6) was dissected ipsilateral to the sciatic lesion, the relevant spinal cord region where primary neurons synapse with the secondary pain projection neurons. All tissues and blood leukocytes were immediately placed in DNase/RNase/Protease-free microtubes (VWR International; Cat#: 47747–358), frozen on dry ice, and stored at −80°C for future analysis.

Total RNA was isolated using the miRNeasy RNA isolation kit (Qiagen, Hilden, Germany) according to the manufacturer’s instructions, as described previously. Samples were randomly pooled from 2 to 3 biological replicates and 1 ug total RNA material per sample was shipped to Arraystar company under frozen conditions. The following groups were sent for circRNA profiling from the spinal cord: (1) Sac control + Sham surgery, (2) PAE + Sham surgery, (3) Sac control + minor CCI surgery, (4) PAE + minor CCI surgery. For peripheral blood samples, Sac + Sham or PAE + Sham leukocytes were used to examine whether PAE alone modulated basal levels of circRNAs. Individual RNA samples remaining from these tissues were utilized for validating circRNAs and analyzing mRNA levels. Blood RNA samples were purified with Monarch RNA clean-up kit (New England Biolabs, Germany). Due to the limited amount of RNA materials from blood leukocytes, additional blood leukocyte samples were collected from age-matched PAE and Sac rats, without any nerve injury.

### 2.6. CircRNA expression profiling from peripheral leukocytes and spinal cord

CircRNA expression was profiled using Arraystar Rat Circular RNA Microarray service (Arraystar Inc., Rockville, MD). The circRNA array platform consisted of 14,145 probes designed by the manufacturer to detect unique circular junction-specific probes and quantify circular RNA targets at high sensitivity and specificity (Guo et al., 2014; Paudel et al., 2020). Briefly, samples were quantified using a NanoDrop ND-1000. The integrity of RNA samples was assessed by electrophoresis on a denaturing agarose gel. 1 ug of extracted total RNA was treated with RnaseR (3 h at 37°C of ribonuclease R, 20 U/μL, Epicenter, Madison WI) to digest linear RNA and enrich circRNA. Random primers were then used to amplify and transcribe the enriched circRNAs into fluorescent cRNAs per the Arraystar Super RNA Labelling protocol (Arraystar Inc.). Using Arraystar Rat Circular RNA arrays, the labeled cRNAs were hybridized and incubated in an Agilent hybridization oven for 17 h at 65°C (Agilent Technologies, Santa Clara, CA). The slides were washed and scanned using the Agilent Scanner G2505C (Agilent Technologies). The array image was analyzed, and quantile normalization, and subsequent data processing were performed using the LIMMA (implemented with the R software package). Both raw and normalized circRNA levels were then compared to identify differentially altered circRNAs. Heatmap and hierarchical cluster analysis were used to demonstrate the expression patterns of these differentially expressed circRNAs. All these datasets are made publicly available (see below).

### 2.7. Quantification of circRNA and mRNA expression

CircRNAs were validated with custom-designed primers to amplify back-spliced junctions specific to circRNA, as described in prior reports from our group, Mellios (Paudel et al., 2020; Zimmerman et al., 2020) and (Dell'Orco et al., 2020). Briefly, reverse transcription was performed using the SuperScript IV First-Strand Synthesis System (ThermoFisher Scientific) with random hexamers for circRNA and separately, with oligo-dT primers for linear mRNA detection. For circRNA detection, cDNA was then used together with custom-made, validated, and sequence-verified circRNA primers and PowerUp SYBR green master mix (Thermo Fisher Scientific). The following formula was used to calculate normalized or relative values of circRNAs: Relative value = 2^Ct-normalizer/A^Ct-circRNA, where *A* = 10^ (−1/primer slope). All circRNA primers were tested for their resistance to RNase R and reduced abundance in oligo-dT reverse-transcribed cDNA, together with melting curve and primer slope analysis, as described in prior studies (Paudel et al., 2020; Zimmerman et al., 2020). For qRT-PCR quantification for mRNA, commercially available TaqMan probes were used, as described in our prior reports (Vanderwall et al., 2017; Noor et al., 2020). The following formula was used: Relative value = 2^Ct-normalizer/2^Ct-mRNA. 18 s ribosomal RNA or GAPDH was used as a normalizer. All samples were run in triplicates. Data were acquired and melt curves were analyzed using QuantStudio 7 Flex system (Themofisher Scientific, United States).

### 2.8. Ingenuity pathway analysis (IPA)

For comparisons, normalized circRNA expression levels that were significantly altered (>1.5-fold, *p* < 0.05) were used for further bioinformatic analyses (see detail below) to identify potential immune or neuroimmune regulatory effects of the genes that generate these circRNAs. Ingenuity Pathway Analysis (IPA) (Krämer et al., 2014) online software (IPA, QIAGEN Redwood City, CA, United States) was employed to conduct unbiased Core Analysis using the Arraystar microarray data (Arraystar Inc.). IPA identified canonical signaling pathways and their categories, associated diseases and functions, and top molecular networks associated with these genes. Gene IDs associated with significantly dysregulated circRNAs and the fold changes (“+” indicating upregulation, “−” indicating downregulation) were included for IPA analysis. IPA analysis calculated the *p*-values using a right-tailed Fisher’s Exact Test. The *p*-values ≤0.05 (−log = 1.3) indicated a statistically significant, non-random association with the molecules from the dataset being compared with the IPA-listed pathways or diseases and functions. The z-score provides the direction and magnitude of the enrichment. An absolute z-score ≥ 2 or ≤ −2 was considered significant.

### 2.9. Statistical analysis

SPSS (IBM, Chicago, IL, United States) or GraphPad Prism was used for statistical analyses. For behavioral data analysis, at baseline (BL), a 2-way (2 × 2) analysis of variance (ANOVA) to assess differences between prenatal exposure (Sac vs. PAE) and surgical treatment (Sham vs. minor CCI). Additionally, a 2-way (2 × 2) repeated measures ANOVA was used for the analysis of the between-subject factors of prenatal exposure, surgery, and for days post-surgery. To control the type I error rate, Tukey’s test was applied for *post hoc* examination and adjusted *p*-values are reported. mRNA and circRNA levels were analyzed using a 2-way ANOVA, with Tukey’s test for multiple group comparisons. For comparisons between the two groups, unpaired *t*-tests were performed. The threshold for statistical significance was set *a priori* at *p* < 0.05 for all comparisons. Outliers were removed following Grubbs’ *Z*-test (Grubbs, 1950). In all cases, the data are presented as the mean ± SEM. All figures are created in GraphPad Prism version 9 software (GraphPad Software Inc., San Diego, CA, United States).

## 3. Results

### 3.1. Paradigm outcome measures

Rat dams in the 5% ethanol group consumed a mean of 1.96 ± 0.06 grams of ethanol each day during gestation (Table 1). In a separate set of rat dams, this level of consumption produced a low to moderate level of mean serum ethanol concentration of 25.1 ± 3.3 mg/dL, when sampled 2 h after the introduction of drinking tubes (Davies et al., 2023). This prenatal ethanol exposure paradigm resulted in a 7.5% reduction in maternal weight gain that was statistically significant. However, this reduction in maternal weight gain did not affect offspring litter size or offspring birth weight (Table 1).

**Table 1:** Prenatal alcohol exposure paradigm outcomes.

	Saccharin control	5% ethanol group
Daily 4 h 5% ethanol consumption	NA	1.96 ± 0.06^a^ (23)
Maternal serum ethanol concentration	NA	25.1 ± 3.3^b^ (14)
Maternal weight gain during pregnancy	110.8 ± 2.3^c^ (25)	102.5 ± 3.1* (23)
Litter size	11.1 ± 0.5^d^ (25)	11.0 ± 0.4 (23)
Pup birth weight	8.22 ± 0.32^e^ (25)	7.95 ± 0.27 (23)

a Mean ± S.E.M. grams ethanol consumed/kg body weight/day.

b Mean ± S.E.M. mg ethanol/dL in serum. Serum samples were collected 2 h after the introduction of the drinking tubes from a separate set of rat dams. This time point is at or near the peak blood ethanol concentration as ethanol consumption reduces significantly in the third and fourth hours of the drinking session (Davies et al., 2023).

c Mean ± S.E.M. grams increase in body weight from GD 1 through GD 21. Asterisk denotes maternal weight gain data significantly less than control rats (*t* = 2.10, *p* = 0.042).

d Mean ± S.E.M. number of live births/litter.

e Mean ± S.E.M. grams pup birth weight.

NA, not applicable, (*n*)-Group sample.

### 3.2. A minor nerve injury model to unmask PAE-induced susceptibility to chronic allodynia

**Figure 1:**
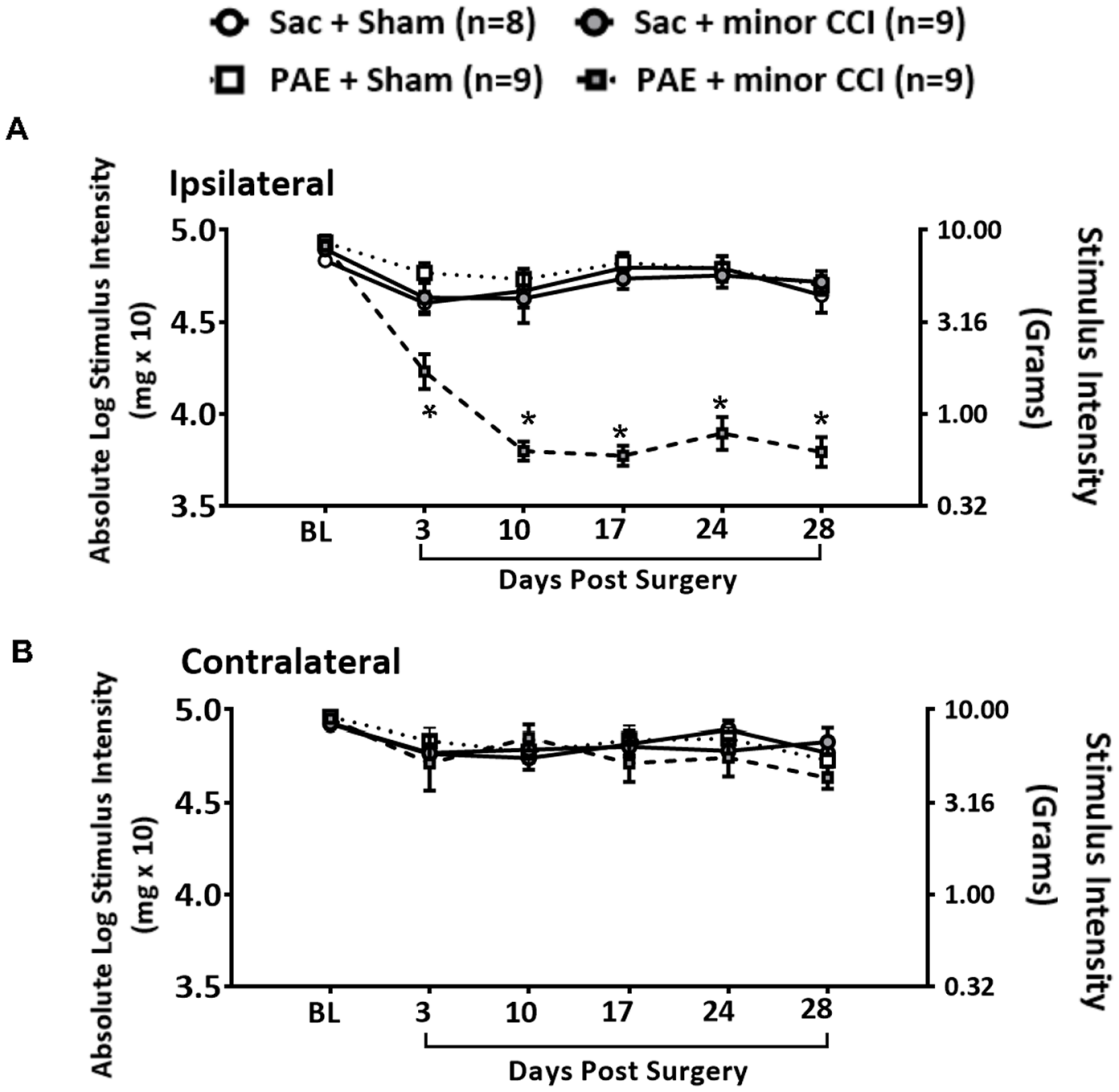
A minor chronic constriction injury (CCI) establishes chronic allodynia in PAE rats. Age-matched adult Sac and PAE rats were subjected to a minor sciatic nerve chronic constriction injury (CCI) to reveal the PAE-induced risk of neuropathy. Hindpaw light touch thresholds were measured using calibrated monofilaments applied to the planter hindpaw. **(A)** Hindpaw responses of PAE vs. Sac control rats showed no significant differences at the pre-surgery baseline (BL) in the ipsilateral hindpaw (*F*_3,31_ = 2.587, *p* = 0.071). Following minor CCI, a main effect of prenatal exposure (ipsilateral, *F*_1,31_ = 42.477, *p* < 0.001) and surgery (ipsilateral, *F*_1,31_ = 69.783, *p* < 0.001) and an interaction between prenatal exposure and surgery (ipsilateral, *F*_1,31_ = 77.001, *p* < 0.001) was observed in the ipsilateral hindpaw. Hindpaw responses to light touch were significantly increased in PAE + minor CCI group compared to Sac + minor CCI group at D3 (*p* = 0.003), D10 through D28 (*p* < 0.0001). *Denotes *p* values with significance. **(B)** For contralateral hindpaw responses, no effect of prenatal exposure and nerve injury at any time point was observed. Error bars represent SEM (standard error of mean). Number of rats used: *N* = 8 in Sac + Sham group, Sac + minor CCI, PAE + Sham, PAE + minor CCI, *N* = 9 in each group.

A minor nerve injury model was utilized to unmask the susceptibility to develop allodynia in PAE adult offspring. Under baseline conditions (BL), light touch sensory thresholds demonstrated no significant differences in all rat groups, with responses occurring at approximately 10 g of touch stimuli (Figures 1A,B). Following Sham surgery, non-PAE control (Sac) and PAE groups maintained normal sensitivity throughout the time course in the ipsilateral and contralateral (opposite hindpaw of the site of nerve injury site) hindpaws. These data suggest that PAE without injury, did not alter hindpaw sensitivity. Following a minor sciatic nerve injury, ipsilateral hindpaw responses remained close to their BL values in Sac rats, with sensory thresholds measured on all post-CCI time-points overlapping with Sham-injured rats. However, following this minor nerve injury, striking allodynia was observed only in PAE rats, as measured by increased sensitivity of the ipsilateral hindpaw developed by Day 3 post-CCI and displayed persistent allodynia up to 28 days-post CCI (Figure 1A). Contralateral hindpaw responses remained stably near BL values (Figure 1B) in all Sham and minor-nerve injured rats, regardless of prenatal exposure treatment. These data replicate our prior study in adult female PAE rat offspring with minor CCI (Noor et al., 2020) supporting that the role of proinflammatory spinal glia and peripheral immune activation under PAE conditions are key factors that lead to chronic allodynia. These data demonstrate that the persistent insidious effects of prenatal alcohol exposure, even at low to moderate levels, on immune priming are unmasked following a minor challenge. Following testing on Day 28 after induction of CCI, tissues were collected to examine circRNA expression during allodynia in these PAE rats. Samples from Sham-injured rats served as control tissue to compare against the circRNA changes modulated by PAE, as described in sections 3.3 and 3.6.

### 3.3. PAE causes long-term alterations in spinal cord circRNA expression

**Figure 2:**
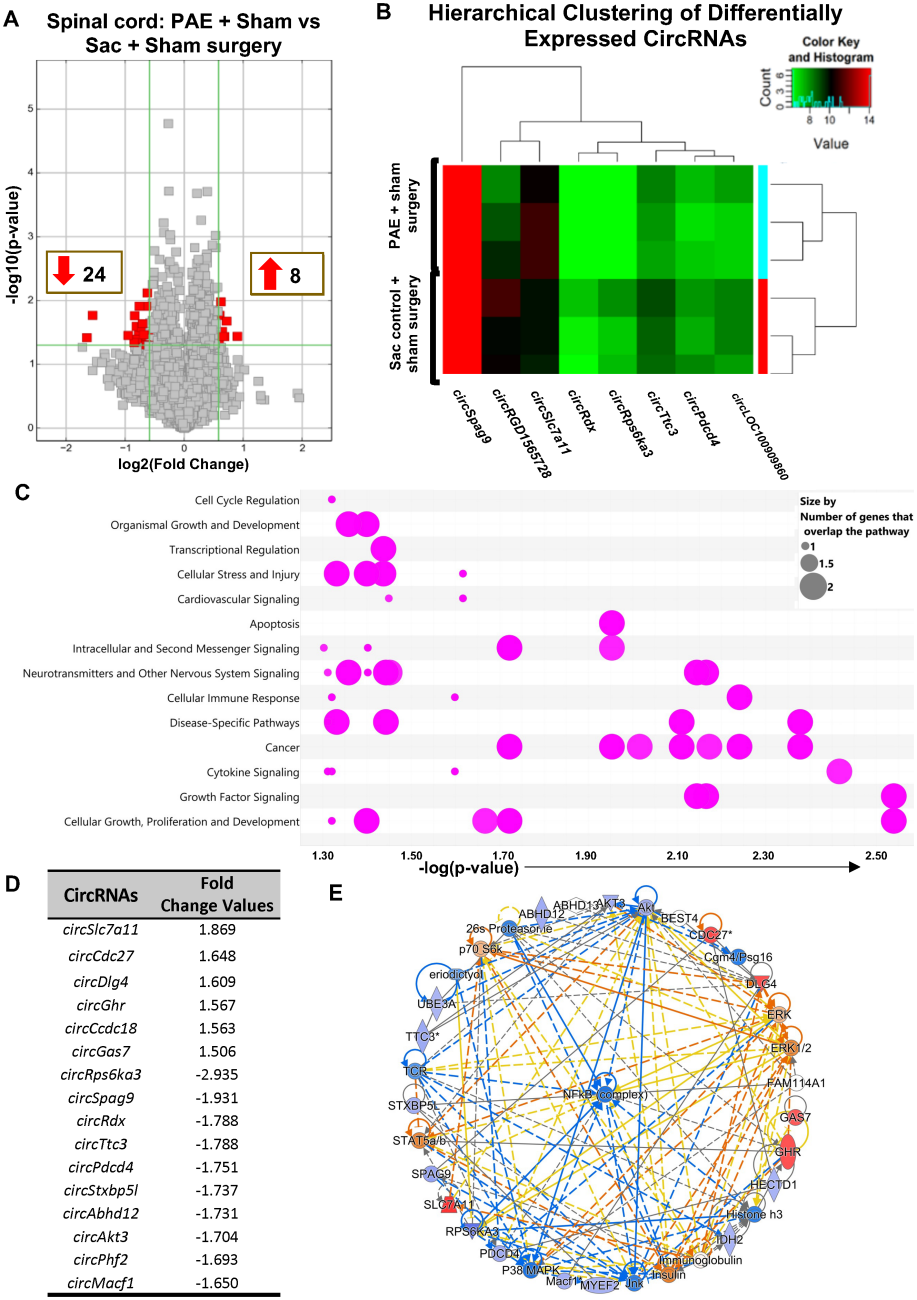
PAE results in altered spinal circRNAs from genes related to the proinflammatory transcription factor NF-κB in the absence of chronic neuropathy. **(A)** Volcano plots displaying differentially expressed circRNAs between PAE and Sac spinal cord, without nerve injury. X-axis represents log2 fold change (dotted line = 1.5-fold), Y-axis represents −log10 *p*-value (dotted line: *p* = 0.05). The red points in the plot represent the differentially expressed (both upregulated and downregulated) circRNAs with 1.5-fold changes (*p* < 0.05). **(B)** Heat map of the circRNA microarray profiles (selected for fold changes of 1.75 or more) in the PAE + Sham surgery and Sac control + Sham surgery groups. The expression of circRNAs is hierarchically clustered on FIGURE 2 (Continued)the Y-axis, and spinal cord biological replicates from these two groups are hierarchically clustered on the X-axis. Green and red represent lower higher expression levels of the circRNAs, respectively. **(C)** Ingenuity Pathway Analysis (IPA) of host genes associated with differentially expressed circRNAs. The bubble chart plots the pathway categories (Y-axis) related to these genes vs. the negative log of the *p*-values (X-axis), the smaller the *p*-values (a larger −log of that value) indicates a more significant association. This plot demonstrates the major functional categories of various associated canonical signaling pathways (as indicated by bubbles) regulating critical functions such as cell cycle regulation, cellular stress and injury, neurotransmitters and other nervous system signaling, and cytokine signaling, amongst many more. The associations with signaling pathways are selected for IPA analysis (pink color), the size refers to the number of genes that overlap with a pathway. **(D)** Top upregulated and downregulated circRNAs, parental or host gene symbol and fold changes, the absolute ratio of normalized intensities between two conditions. **(E)** The comprehensive molecular networks are generated using IPA Canonical Pathways’ top network function. The network comparing PAE to the control group in the spinal cord is generated from the primary top network (z-score = 47). Molecules in blue and red are downregulated and upregulated, respectively. Three different biological replicates (each sample pooled from 2 to 3 rats per group, from Figure 1) per treatment group were used to generate the circRNA microarray data.

CircRNA microarray profiles in non-injured PAE and Sac rats were compared to explore whether PAE influenced the basal levels of circRNAs. Data suggested that more than 30 circRNAs were differentially expressed due to PAE. 24 circRNAs were found downregulated and 8 circRNAs were upregulated in PAE spinal cord, displayed as colored dots in Figure 2A. A heatmap expression pattern was generated displaying selective differentially expressed circRNAs, up- or down-regulated by a factor of greater than 1.75 (Figure 2B). Using IPA, an unbiased approach was taken to determine the pathways and molecular networks associated with these parental genes, with a cut-off at 1.5-fold change with *p* < 0.05, including both up-regulated and down-regulated circRNAs. This bioinformatics analysis platform suggested that these genes were related to canonical cellular signaling pathways regulating critical CNS and immune functions including transcriptional regulation, cytokine and neurotransmitter signaling, cellular stress and injury, and cellular immune responses (Figure 2C and Supplementary Figure S1E, the list of the top 5 pathways). IPA analysis also detected top up- or down-regulated circRNAs, with a cut-off value of 1.5 (Figure 2D). Additionally, “top networks” of the genes associated with these differentially expressed circRNAs were analyzed. Interestingly, this analysis demonstrated that the top molecular network of parental genes was related to a key transcription factor, nuclear factor-kappa B (NF-kB), which is a central driver for the transcription of several pain-relevant proinflammatory cytokines (Figure 2E).

### 3.4. Minor nerve injury results in an overlapping yet distinct circRNA expression profile in PAE rats compared to control rats

**Figure 3:**
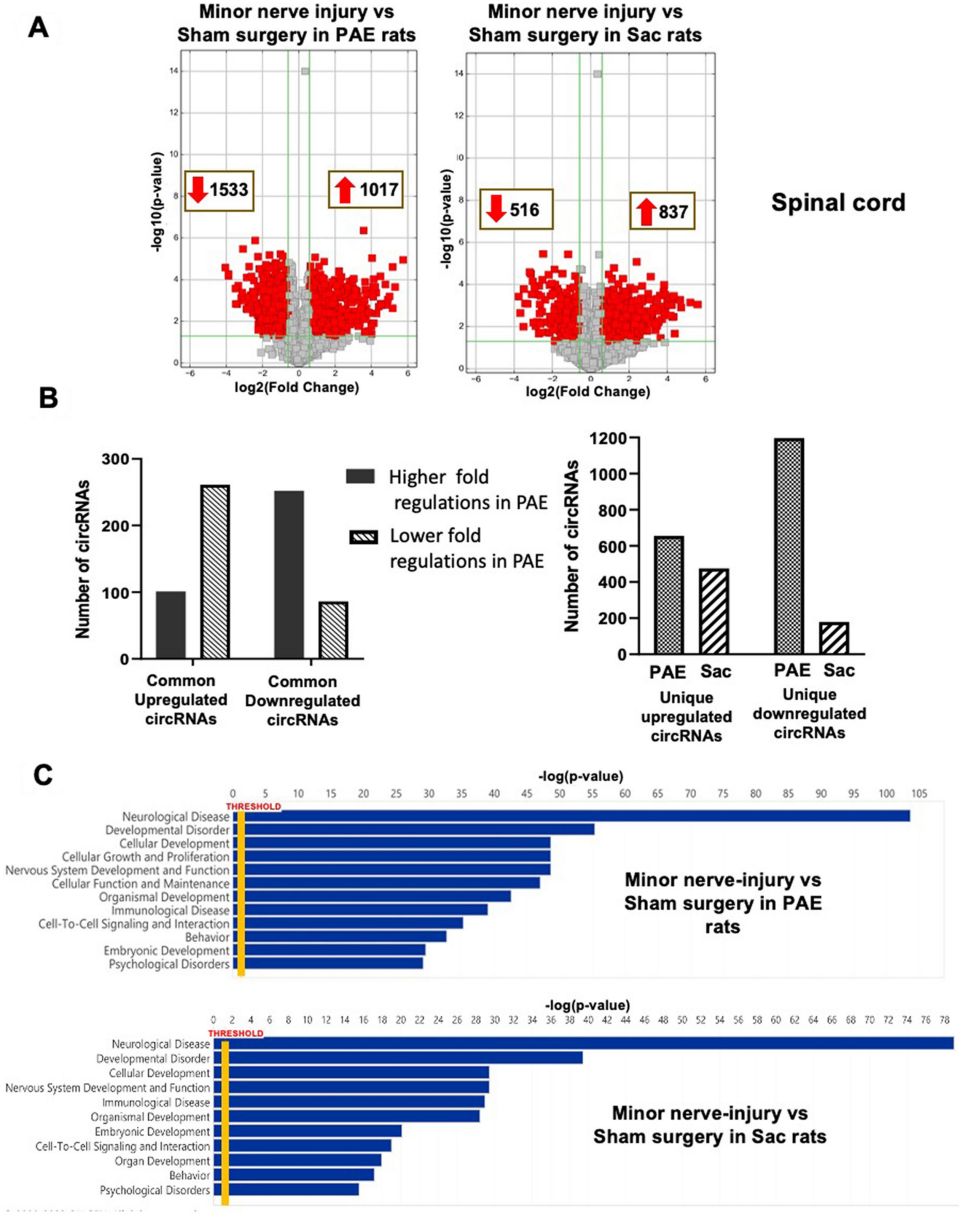
PAE differentially modulates spinal circRNA expression following a minor nerve injury. **(A)** Volcano plots of differentially expressed circRNAs between nerve-injured PAE and Sac spinal cord, compared to their corresponding control tissues. X-axis represents log2 fold change (dotted line = 1.5-fold), Y-axis represents –log10 *p-*value (dotted line: *p* = 0.05). The red points in the plot represent the differentially expressed circRNAs, with 1.5-fold changes (*p* < 0.05). **(B)** The bar graphs represent the number of differentially expressed circRNAs as a result of injury, that are common or unique in PAE and Sac control rats. Among the altered circRNAs that are common in Sac control and PAE rats, the numbers of circRNAs that are further up or down-regulated in PAE conditions as a result of nerve injury are shown. **(C)** Ingenuity Pathway Analysis (IPA) from the host genes of the differentially expressed circRNAs is used to generate the list of associated diseases or functions (Y-axis) with associated −log *p*-values (X-axis).

The effect of minor nerve injury on spinal cord circRNA expression profile was examined in Sac and PAE rats separately in comparison to their corresponding sham-surgery controls. Minor nerve injury dysregulated more than a thousand circRNAs from spinal cords of either Sac or PAE rats. Minor nerve injury upregulated 837 circRNAs in Sac rats and 1,017 circRNAs in PAE rats. Additionally, minor nerve injury downregulated 516 circRNAs in Sac rats and 1,533 circRNAs in PAE rats (Figure 3A). However, only about 350 circRNAs were common in both Sac and PAE rats that were either upregulated or downregulated, as a result of nerve injury (Figure 3B). Comparing the ratio of the fold regulations of these circRNAs that were common in these two datasets, about 27% of the circRNAs that were upregulated in Sac rats, were further upregulated in PAE rats. Among the commonly downregulated circRNAs following minor never injury, more than 70% circRNAs revealed a more pronounced downregulation in PAE rats (Figure 3B). Additionally, more than a thousand differentially regulated circRNAs were detected in PAE rats, which were not modulated in nerve-injured Sac rats. These data suggested that PAE not only influenced the fold regulations of circRNAs that were dysregulated by nerve injury in control conditions, but PAE also generated unique circRNA changes as a result of nerve injury. IPA analysis of the circRNA host genes in nerve-injured Sac rats and nerve-injured PAE rats detected relevance to multiple diseases and biological functions in common, such as neurological diseases, developmental disorders, immunological diseases, cell-to-cell interactions and psychological disorders. However, an overall stronger association, with higher −log (*p*-values), was observed in PAE rats (Figure 3C). The top 50 canonical signaling pathways associated with CNS function were compared between minor nerve injury-induced circRNAs in PAE vs. Sac rats (Tables 2, 3). In both groups, a number of common and pain-relevant pathways, such as in dorsal horn neurons and glutamate receptor signaling or circadian rhythm signaling, were associated with these genes. However, increased −log (*p*-values) indicated a stronger association with these pathways in PAE rats (Table 2). Additionally, G-protein receptor signaling, neurovascular coupling, corticotropin-releasing hormone signaling, chemokine CXCR4 signaling were noted among the top 50 pathways only in PAE rats, indicating a stronger association of these pathways under PAE conditions. The top 50 pathways in Sac rats included PPAR signaling, integrin, and JAK/STAT signaling pathways. Although these pathways were not in the top 50 associated pathways in PAE rats, a similar or weaker association (lower −log *p*-values) of these pathways was observed (Table 3). Together, these data suggest that following minor nerve injury, differentially regulated circRNAs are derived from genes that are highly associated with a number of common pathways in both Sac and PAE conditions. However, a number of additional canonical signaling pathways are found to be strongly associated with circRNA changes in PAE rats.

**Table 2:** Common canonical pathways associated with circRNA host genes in both PAE and Sac rats following minor nerve injury.

Ingenuity canonical pathways that are included in the top 50 pathways in both PAE and Sac rats	−log(*p*-value), minor nerve injury vs. sham surgery in PAE rats	−log(*p*-value), minor nerve injury vs. sham surgery in Sac rats
Synaptogenesis signaling pathway	16	9.58
Protein kinase A signaling	11.7	4.42
Opioid signaling pathway	10.1	4.53
Neuropathic pain signaling in dorsal horn neurons	6.05	3
Circadian rhythm signaling	9.14	2.76
cAMP-mediated signaling	6.7	3.04
Amyotrophic lateral sclerosis signaling	10.3	6.77
Gap junction signaling	4.64	4
TR/RXR activation	7.44	6.56
Dopamine-DARPP32 feedback in cAMP signaling	9.45	4.89
Insulin secretion signaling pathway	5.66	3.45
Calcium signaling	10.8	4.81
CLEAR signaling pathway	5.6	4.85
Glutamate receptor signaling	8.58	3.94
Myelination signaling pathway	5.35	3.1
Synaptic long-term potentiation	7.62	4.67
Autophagy	6.17	5.46
Adrenomedullin signaling pathway	4.17	3.5
Molecular mechanisms of cancer	5.64	4.39
NGF signaling	4.32	2.89
Oxytocin signaling pathway	5.29	3.63
IL-15 production	6.39	3.87
Sphingosine-1-phosphate signaling	4.32	3.42
PPARα/RXRα activation	4.32	6.23
Endocannabinoid neuronal synapse pathway	8.92	5.76
Synaptic long-term depression	7.05	2.66
Estrogen receptor signaling	6.9	4.85
Huntington’s disease signaling	5.67	4.46
Axonal guidance signaling	5.39	4.02
SNARE signaling pathway	5.06	3.41
RAR activation	4.8	4.78
Senescence pathway	4.36	2.9
Ephrin A signaling	4.29	3.62

**Table 3:** List of other top 50 pathways associated with circRNA host genes in either PAE or Sac rats following minor nerve injury.

	−log(*p*-value)
**Ingenuity canonical pathways in minor-nerve injury vs. sham surgery in PAE rats**	
Neurovascular coupling signaling pathway	7.38
Apelin endothelial signaling pathway	4.31
Netrin signaling	8.69
GPCR-mediated nutrient sensing in enteroendocrine cells	5.53
Endocannabinoid cancer inhibition pathway	4.51
GABA receptor signaling	5.27
CXCR4 signaling	4.07
Epithelial adherens junction signaling	5.03
GNRH signaling	5.87
Corticotropin releasing hormone signaling	6.9
Reelin signaling in neurons	5.48
Gα12/13 signaling	5.22
Gαq signaling	4.44
Semaphorin neuronal repulsive signaling pathway	3.91
Cholecystokinin/gastrin-mediated signaling	3.87
Type II diabetes mellitus signaling	3.79
Relaxin signaling	3.71
**Ingenuity canonical pathways in minor nerve injury vs. sham surgery in Sac rats**	
GM-CSF signaling	3.72
nNOS signaling in neurons	3.62
Renin-angiotensin signaling	3.38
Glioblastoma multiforme signaling	3.34
PPAR signaling	3.34
HIF1α signaling	3.27
Integrin signaling	3.17
Ephrin receptor signaling	2.99
Insulin receptor signaling	2.79
ERBB signaling	2.73
IL-3 signaling	2.69
Serotonin receptor signaling	1.66
IL-2 signaling	1.63
IL-23 signaling pathway	1.62
JAK/STAT signaling	1.56
D-myo-inositol (1,4,5,6)-tetrakisphosphate biosynthesis	1.55
D-myo-inositol (3,4,5,6)-tetrakisphosphate biosynthesis	1.55

### 3.5. Significantly differentially expressed circRNAs are observed between nerve-injured sac and PAE rats

**Figure 4:**
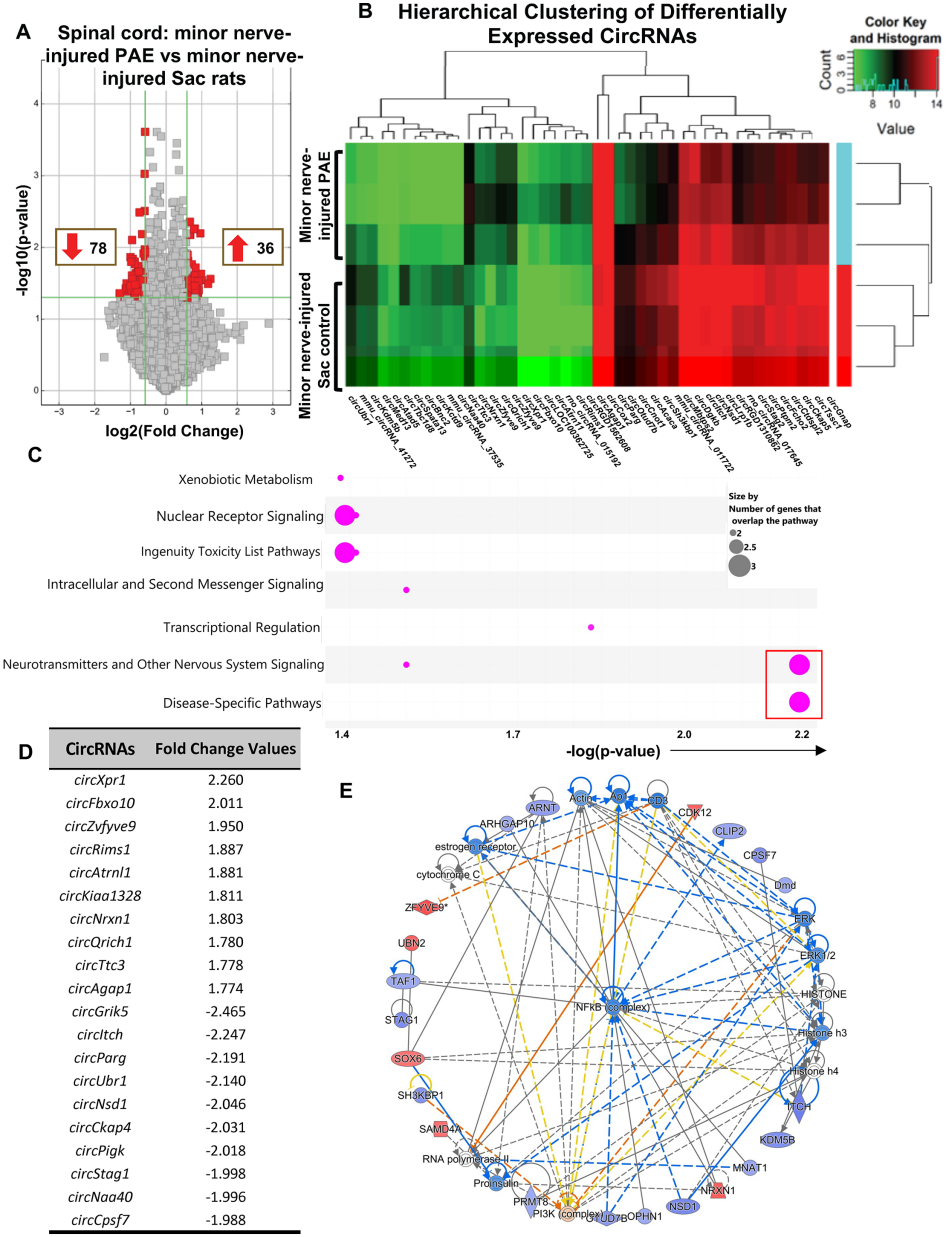
Differential circRNA expression was observed in the spinal cord in PAE rats during allodynia, compared to non-allodynic control rats. **(A)** Volcano plots of differentially expressed circRNAs between PAE and Sac spinal cord, following nerve injury. X-axis represents log2 fold change (dotted line = 1.5-fold), Y-axis represents –log10 *p-*value (dotted line: *p* = 0.05). The red points in the plot represent the differentially expressed circRNAs, with 1.5-fold changes (*p* < 0.05). **(B)** Heat map of the circRNA microarray profiles in the minor nerve-injured PAE (allodynic) and minor nerve-injured Sac control groups (non-allodynic), more than 1.75-fold changes. The expression of circRNAs is hierarchically clustered on the Y-axis, and spinal cord samples are hierarchically clustered on the X-axis. Green and red represent lower and higher expression levels of the circRNAs. **(C)** Ingenuity Pathway Analysis (IPA) from the host FIGURE 4 (Continued)genes of the differentially expressed circRNAs is used to generate the bubble plot. The bubble chart plots the pathway categories (Y-axis) related to these genes vs. the negative log of the *p*-values (X-axis), demonstrating the involvement of these genes with important canonical pathway categories such as nuclear receptor signaling, neurotransmitters and nervous system signaling and disease-specific pathways. The red square indicates bubbles referring to the neuropathic pain signaling pathway as the most significantly associated pathway. **(D)** Top upregulated and downregulated spinal circRNAs in minor nerve injured PAE rats compared to minor nerve injured control rats, parental or host gene symbol and fold changes with a cut off at 1.5-fold. **(E)** The three comprehensive networks are generated using IPA Canonical Pathways’ top network function. The network compares minor nerve-injured PAE to minor nerve-injured control in the spinal cord and uses the secondary top network (z-score = 46). Molecules in blue and red are downregulated and upregulated, respectively. Three separate biological replicates per treatment group were used to generate these data.

To explore minor nerve injury-induced differential pattern of circRNA expression in PAE rats, which may be linked to key modulators of PAE-related aberrant immune interactions causing allodynia (Figure 1A), circRNA profiles in the nerve-inured PAE (allodynic) vs. nerve-injured Sac rats (non-allodynic) were compared. Data revealed that 36 circRNAs were significantly up-regulated and 78 circRNAs significantly down-regulated in PAE spinal cord compared to minor-nerve injured Sac rats (Figure 4A). A clustered heatmap exhibited a differential pattern of spinal circRNAs between these two groups of rats (Figure 4B). The top 10 up- or down-regulated circRNAs were listed in Figure 4D. IPA was utilized to determine the signaling pathways and molecular networks associated with these parental genes, with a cut-off at 1.5-fold change with *p* < 0.05, including both up-regulated and down-regulated circRNAs. Interestingly, IPA results showed that these differentially expressed circRNAs were preferentially linked to genes related to neuropathic pain signaling, as indicated by the lowest *p*-values for this pathway (Figure 4C; Supplementary Figure S2E). Involvement of other relevant pathways such as nuclear receptor signaling or neurotransmitter signaling and disease-specific pathways and the relation to xenobiotics and ingenuity toxicity pathways were noted. Moreover, focusing on the molecular networks that were formed from these genes, two networks with a z-score greater than 40 suggested relevance to categories such as cell–cell interactions and nervous system function and indicated involvement of these parental genes with the NF-κB immune complex (Figure 4E; Supplementary Figure S2C).

### 3.6. PAE produces a unique signature pattern of circRNAs detected in blood-circulating immune cells

**Figure 5:**
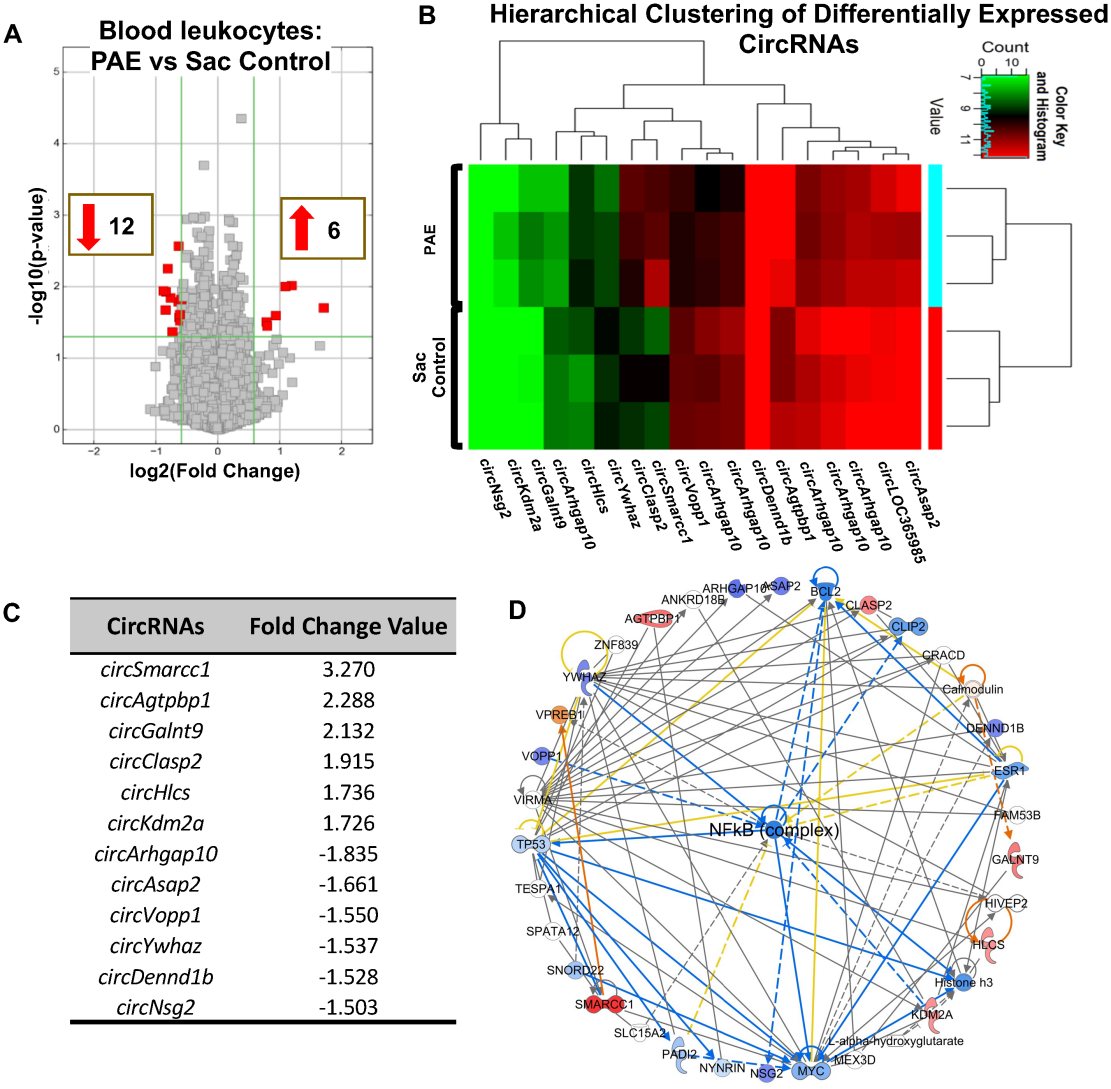
PAE-induced dysregulation of circRNAs is evident in blood leukocytes and is related to NF-κB-associated genes. **(A)** Volcano plots of differentially expressed circRNAs between PAE and Sac blood leukocytes without nerve injury. X-axis represents Log2 fold change (dotted line = 1.5-fold), Y-axis represents –log10 *p*-value (dotted line: *p* = 0.05). The red points in the plot represent the differentially expressed circRNAs, with 1.5-fold changes (*p* < 0.05). **(B)** Heat map of the differentially expressed circRNAs in the PAE and control groups with fold changes of 1.5 or more. The expression of circRNAs is hierarchically clustered on the Y-axis, and biological replicates in different groups are hierarchically clustered on the X-axis. Green represents a lower and red represents a higher expression level of the circRNAs. **(C)** Top upregulated and downregulated circRNAs in PAE vs. Sac control leukocytes **(D)** The comprehensive networks are generated using IPA Canonical Pathways’ top network function. The network graph’s primary top network compares PAE vs. control in the blood leukocyte (z-score = 37). Three separate biological replicates (each sample was pooled from 2 to 3 rats from Figure 1) per treatment group were used to generate these data.

Given substantial evidence that peripheral immune activation contributes to PAE-induced deficits (Bodnar et al., 2016; Terasaki and Schwarz, 2016; Sanchez et al., 2017; Noor et al., 2020; Bake et al., 2021) during adulthood and its potential relevance to biomarker development, we carried out an analysis of circRNAs that are specifically present in peripheral immune cells. Although far fewer in numbers compared to our observations in the spinal cord tissues, circRNAs were detected in the peripheral leukocytes. Comparing these circRNAs between uninjured PAE and Sac rats revealed a unique circRNA signature profile due to PAE (Figures 5A–D). Hierarchical clustering analysis indicated that circRNA expression patterns were distinguishable between PAE and Sac blood leukocytes (Figure 5B). Some of these differentially expressed circRNAs were derived from genes including those associated with glucocorticoid receptor signaling (*Smarcc1*), Rho GTPase activating protein (*Arhgap10*), ATP/GTP binding Carboxypeptidase (*Agtpbp1*) and Vesicular, Overexpressed in Cancer, and Prosurvival Protein 1 (*Vopp1*). CircRNAs were generated from genes involved in cell–cell interactions, immune cell trafficking, inflammatory response, and genes linked to psychological and neurological diseases (Supplementary Figures S3D,E). Notably, no overlap was observed among the differentially expressed circRNAs detected in blood compared to the spinal cord, between PAE and Sac rats under non-injured conditions. Although driven by a different set of host genes observed in blood relative to spinal cord, the top molecular network with the highest z-score from these peripheral circRNA host genes further suggested the involvement of the NF-κB pathway (Figure 5D).

### 3.7. Exploring circRNAs and mRNA levels of host genes potentially relevant to neuroimmune function

**Figure 6:**
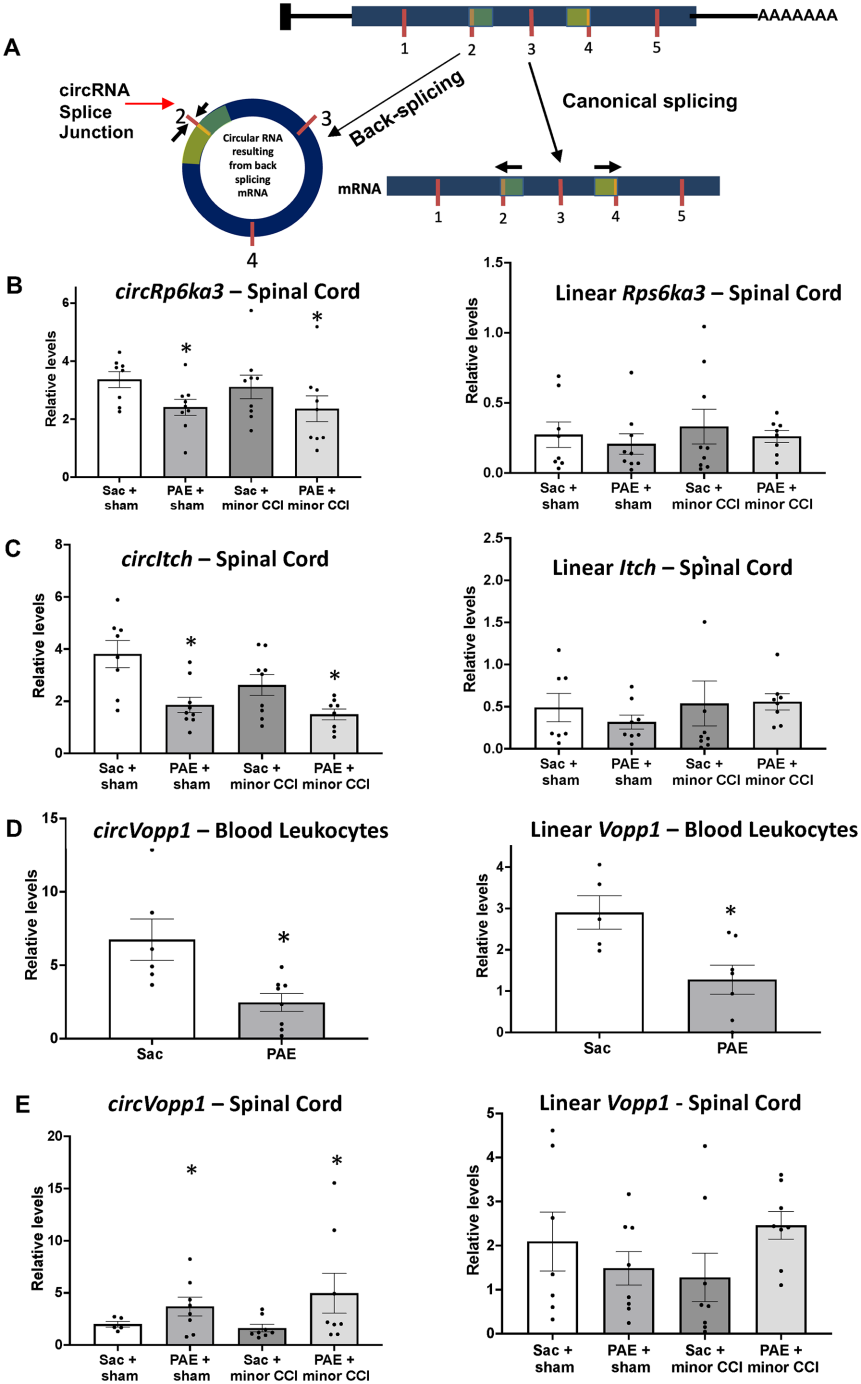
Quantitative real-time PCR validated dysregulated levels of circRNAs and their linear isoforms. **(A)** Schematic representation of an example circRNA biogenesis resulting from back-splicing events of Exon 2 and Exon 4 from a hypothetical precursor mRNA. The divergent primers for circRNA qRT-PCR validation were designed such that only the back-spliced and not linear junction was amplified. **(B)** Spinal *circRps6ka3* and linear *Rps6ka3* levels were measured. A main effect of PAE (*F*_1,31_ = 5.53, *p* = 0.025) was observed in *circRps6ka3* levels. An unpaired t-test revealed a significant downregulation of *circRps6ka3* levels (*p* < 0.03) between PAE vs. Sac control, Sham injured rats. mRNA levels were not different across treatment groups. **(C)** *CircItch* levels were downregulated with PAE, a main effect (*F*_1,29_ = 4.58, *p* = 0.004) was observed. A main effect of surgery (*F*_1,29_ = 15.66, *p* < 0.05) was also observed. Post-doc test revealed a significant difference between PAE to Sac control (*p* < 0.005), Sham-injured groups. Although the levels were not significantlyFIGURE 6 (Continued) different with 2-way ANOVA, an unpaired t-test revealed significant downregulation of *circItch* levels (*p* < 0.05) between nerve-injured PAE vs. nerve injure Sac control rats. mRNA levels were not different across treatment groups. **(D)** Blood leukocytes from additional adult Sac (*N* = 6) and PAE (*N* = 8) female rats were used for validation. A significant decrease of both *circVopp1* (*p* < 0.01) and linear *Vopp1* (*p* < 0.02) levels was observed in PAE. **(E)** A main effect of PAE (*F*_1,25_ = 4.25, *p < 0.05) was observed on *circVopp1* levels in the spinal cord, however, multiple comparisons were not significant between groups. mRNA levels were not different across treatment groups, however, a trend of increased *Vopp1* mRNA levels in nerve-injured PAE rats was observed compared to the nerve-injured Sac rats. *Denotes *p* values with significance.

Based on our circRNA array results and prior studies suggesting NF-κB signaling is a key immune modulator in PAE-related immune sensitization, we speculated that some of these circRNAs may have a direct or indirect influence on the mRNA levels of their corresponding host/parent genes (Figure 6A), hence contributing to proinflammatory bias under PAE conditions observed in our prior studies (Noor et al., 2020). Therefore, we further explored the top molecules (Figures 2D, 4D, 5C) through databases, Gene Cards and Gene, NCBI, and consequently selected some genes to examine their mRNA levels. Selected genes from these differentially regulated circRNAs that are known to contribute to immune function or pathological pain processing are listed in Table 4. Additionally, utilizing custom-designed splice-junction-specific primers, we have validated one circRNA from each comparison category (Figures 2–4). Supporting the microarray data presented in Figure 2, we have validated that *circRp6ka3* levels were down-regulated (Figure 6B) in PAE-Sham spinal cord compared to Sac-Sham spinal cord. Comparing *circItch* levels, we have validated that *circItch* levels were down-regulated (Figure 6C) following nerve injury in PAE rats. However, this downregulation was also observed at the baseline, and a main effect of PAE and surgery was observed. We did not observe changes in mRNA levels of *Itch* or *Rp6ka3* mRNA levels (Figures 6B,C).

**Table 4:** Selected circRNAs arising from genes that are associated with immune signaling and/or allodynia.

CircRNA	Tissue	Comparison Category	Fold changes (up or down)	Role in the genes in immune signaling or pathological pain	References
*CircRps6ka3*	Spinal	PAE vs. Sac, no nerve injury	−2.93	Serine/threonine kinase that phosphorylates various substrates, including members of the mitogen-activated kinase (MAPK) signaling pathway. Related to Activated TLR4 signaling	Kakugawa et al. (2009)
*CircSlc7a11*	Spinal	PAE vs. Sac, no nerve injury	1.86	Inverse relationship of SLC7A11 and inflammatory cytokines are observed during CNS injury	Sprimont et al. (2021)
*CircOtud7b*	Spinal	PAE vs. Sac, nerve injured	−1.82	Negative regulator of the non-canonical NF-κB pathway	Hu et al. (2013)
*CircGrik5*	Spinal	PAE vs. Sac, nerve injured	−2.46	Glutamate-gated ion channel, involved in sensitization in nociceptive neurons	
*CircItch*	Spinal	PAE vs. Sac, nerve injured	−2.24	Ubiquitin ligase, connected to MAPK and Wnt signaling, a negative regulator of NF-κB signaling	Su et al. (2022) and Kong et al. (2020)
*CircFkbp9*	Spinal	PAE vs. Sac, nerve injured	−1.58	FK506-binding protein 9, involved in preventing stress-induced cell death	Gan et al. (2022)
*CircVopp1*	Blood	PAE vs. Sac, no nerve injury	−1.55	Regulates NF-κB1 translocation to nucleus to initiate its transcriptional activity	Park and James, (2005)

To explore circRNA changes due to PAE that could be detected in the peripheral immune system, we prioritized validating *circVopp1* levels based on prior studies of *Vopp1* as a regulator of nuclear translocation of NF-κB (Park and James, 2005). We have validated the levels of *circVopp1* and supporting our array data, *circVopp1* levels were significantly downregulated in PAE leukocytes (Figure 6D) compared to Sac control leukocytes. More interestingly, we found that *Vopp1* linear mRNA levels were also downregulated due to PAE. In contrast to our peripheral leukocyte data, spinal *circVopp1* levels were up-regulated due to PAE (Figure 6E). Although a trend of higher levels (*p* = 0.08) of linear *Vopp1* levels was observed in the PAE spinal cord following minor nerve injury, statistical significance was not observed across the different treatment groups.

## 4. Discussion

To our knowledge, this is the first study to provide preclinical evidence that prenatal alcohol exposure, at low to moderate levels, induces long-lasting modulation of circRNA expression profiles that are detectable during adulthood. Our data suggest that: (1) PAE itself dysregulates circRNAs in peripheral blood leukocytes and in the spinal cord without injury. Alterations in circRNA detected from blood-derived immune cells during adulthood can act as novel biomarkers to identify adult populations with FASD (Lu and Xu, 2016; Liu et al., 2020, 2021; Ravanidis et al., 2021). (2) Dysregulation of circRNAs displays a distinct pattern, which is dependent on tissue/sample type, and is further regulated by subsequent immune insult, as evidenced by the application of a minor nerve injury model. Although minor nerve injury resulted in dysregulated circRNAs that are common in PAE and Sac rats, a unique profile of circRNA modulation was also observed in PAE rats due to minor nerve injury. (3) Our comprehensive analysis of all these datasets from PAE relative to non-PAE, with or without nerve injury, demonstrate that the host genes of these circRNAs are related to NF-κB-related immune signaling. Together with our prior observations, the data are supportive of the idea that targeting NF-κB signaling and downstream immune molecules may be effective for mitigating FASD-related CNS pathologies (Doremus-Fitzwater et al., 2020). (4) Multiple circRNAs that are known to play modulatory roles in neuroimmune function are found to be dysregulated following nerve injury in PAE rats (discussed below). (5) Our data suggest that PAE dysregulates levels of *circVopp1* concurrent with changes in the levels of linear *Vopp1*, supporting *Vopp1* as a potential mechanism of neuroimmune dysfunction under PAE conditions.

Animal models of PAE demonstrate many similar manifestations of FASD that likewise reflect CNS dysfunction (Panczakiewicz et al., 2016; Reid et al., 2021) involving heightened immune reactivity (Topiwala et al., 2017; Himmelrich et al., 2020). Several animal studies support the emerging hypothesis that underlying CNS dysfunction from PAE may not manifest until a second challenge is encountered, such as from adult-onset nerve injury or typical immune stimulation (Terasaki and Schwarz, 2016; Sanchez et al., 2017). Our prior reports compared the effects of adult-onset nerve injury utilizing both standard CCI (4 sutures) and minor (a single suture) CCI models in PAE rats (Noor et al., 2017; Sanchez et al., 2017). Allodynia in PAE rats was found to be positively correlated with the degree of CCI; that is the standard CCI resulted in much greater allodynia compared to the minor CCI in PAE rats. Additionally, the degree of spinal cord astrocyte and microglial activation increased as the level of sciatic nerve damage increased, from a minor CCI to a standard CCI (Noor et al., 2017; Sanchez et al., 2017). Our prior published reports support that the underlying mechanism that causes a risk of developing allodynia in PAE rats is linked to heightened neuroimmune signaling (Sanchez et al., 2019). Specifically, the role of the proinflammatory cytokine, IL-1β, was critical in the maintenance of neuropathy in adult rats with PAE and minor injury. Together, both standard CCI and minor CCI model is expected to involve similar pathophysiology of perineural inflammation driving the spinal glial-immune activation and neuronal sensitization leading to chronic allodynia. However, due to heightened immune response as a consequence of PAE, only PAE rats but not the control rats, demonstrate allodynia despite a reduction of the degree of nerve damage with the minor CCI model. Other models of neuropathic pain caused by peripheral nerve injury, such as sciatic nerve ligation or sciatic nerve transection, or spared nerve injury do not allow the experimenter to systematically reduce the injury to a minor insult. Consequently, exploring susceptibility to developing neuropathy in PAE rats is not readily attainable when utilizing these other models. In addition, other suture materials, such as silk sutures that induce peri-neural inflammation may cause increased susceptibility to develop allodynia under PAE conditions, although different onset, magnitude and resolution of allodynia may occur (Dowdall et al., 2005; Grace et al., 2010).

The underlying molecular regulators and key immune pathways driving neuroimmune dysfunction upon adult-inset immune challenges are still under investigation. The well-characterized immune cell surface receptor, toll-like 4 (TLR4) is emerging as the nexus for immune dysregulation from PAE that impacts CNS function (Terasaki and Schwarz, 2016; Pascual et al., 2017) despite that spinal TLR4 gene expression remained unaltered from PAE (Noor et al., 2020). These observations suggest that other regulators may be involved underlying this heightened TLR4 signaling. While speculative, PAE may create a “silent” immune sensitization via changes in circRNA expression in the peripheral immune system and in the CNS. The immune sensitization by alterations in specific circRNAs may act as a “priming” factor that precipitates expression of exaggerated TLR4-related mRNA and protein only after the secondary challenge (e.g., a minor nerve injury) underlying heightened CNS glia and peripheral immune responses as a long-term consequence of PAE (Chen, 2020; Chen et al., 2021). One interesting aspect of this study is the compelling evidence of dysregulated circRNAs preferentially arising from genes that are linked to NF-κB immune complex, which is one of the most studied downstream regulators of the TLR4 signaling pathway. NF-κB is also a downstream effector of other proinflammatory cytokine receptor signaling, such as signaling through IL-1β and TNF-α receptors (Verstrepen et al., 2008; Mitchell et al., 2016). CircRNA biogenesis is paired with active transcription of their parental genes. Thus, these data support prior transcriptome studies in alcohol and PAE literature suggesting NF-κB-responsive genes are potential key modulators of PAE-related CNS dysfunction. Moreover, these bioinformatic analyses of these parental genes provided new NF-κB-related target genes that may contribute to long-term neuroimmune dysregulation due to PAE.

Additionally, our data identified a discrete subset of differentially expressed spinal circRNAs following minor nerve injury. Among these differentially expressed circRNAs are some circRNAs that are proven to be critical regulators of immune and neuroimmune function by recent studies. To be noted, we have found that *circItch* (Liu et al., 2022; Su et al., 2022) is downregulated in PAE spinal cord following nerve injury. Interestingly, *circItch* has been characterized to be involved in several biological functions such as regulating cell proliferation, inflammation, and neovascularization. *CircItch* is proven to act as an anti-inflammatory agent in humans. *Circ-Itch* regulates proinflammatory cytokines such as IL-1β and TNF-α *via* regulating mRNA or *via* interacting with miRNAs (Liu et al., 2022; Su et al., 2022). *CircItch* levels are downregulated in multiple disease models and overexpression of *CircItch* can relieve proinflammatory cytokines in an intervertebral disc degeneration model (Kong et al., 2020). Additionally, *CircItch* may regulate drug resistance or toxicity which could be an area of interest to study the differential effects of drugs under PAE conditions (Hausknecht et al., 2017; Rangmar et al., 2017; Cantacorps et al., 2020). We have also detected altered levels of circRNAs such as *circTttc3* which has been implicated in playing modulatory roles in endogenous stress response or CNS injury (Ma et al., 2021; Yang et al., 2021). Based on these data, our future studies will focus on specific manipulations of circRNA expression *via* knockdown or overexpression studies to determine the necessary roles of these circRNAs underlying PAE-related chronic CNS dysfunction.

CircRNAs and their putative function is an emerging area of research. CircRNAs can regulate gene expression *via* multiple and diverse mechanisms such as interacting with RNA binding proteins and sponging miRNAs (Barrett and Salzman, 2016; Chen, 2020; Chen et al., 2021). Dysregulation of circRNAs can occur with up-regulation, down-regulation, or no change in the host gene expression (Wang et al., 2020; Xu et al., 2020; Shao et al., 2021). A majority of the host gene mRNA levels we have examined in this study appear similar across the different treatment groups suggesting that these circRNAs may act *via* other mechanisms such as interacting with pain-relevant miRNA or other mRNA molecules. Interestingly, we have found that linear levels of *Vopp1* and *circVopp1* are dysregulated by PAE. Interpretation of whether or how *circVopp1* regulates linear *Vopp1* levels is limited at this point, our data suggest that dysregulated circ- and linear *Vopp1* levels may contribute to aberrant NF-κB signaling affecting immune function in PAE offspring. Moreover, peripheral blood-identified circRNAs may serve as potential biomarkers for CNS pathologies (Lu and Xu, 2016; Liu et al., 2020; Piscopo et al., 2022), often an indicator of disease prognosis (Gaffo et al., 2019; Ravanidis et al., 2021). Although opposite to what was observed in blood, our real-time PCR data suggesting the altered *circVopp1* levels in both the periphery and CNS tissue may identify *circVopp1* as an ideal marker of PAE-related CNS dysfunction. Although a minimal overlap of PAE-induced circular RNA molecules was observed in the periphery and in the spinal cord in the absence of injury, differentially expressed circRNAs may overlap in the spinal cord and in the peripheral blood samples following subsequent immune challenges. Future studies from clinical samples will address whether these newly identified blood leukocyte-derived circRNAs are viable biomarkers for FASD populations.

Overall, this study provides preclinical evidence that PAE results in dysregulated circRNAs during adulthood. These data demonstrate that PAE-induced neuropathic pain susceptibility is concurrent with an altered profile of circular RNA expression. Data presented here encourage the scientific premise of studying circRNAs as potential clinical biomarkers and as novel therapeutic targets in adults with FASD. Considering only a limited number of studies focused on utilizing female rodents and our prior knowledge on associated proinflammatory glia and peripheral immune factors in minor nerve-injured PAE female rats (Noor et al., 2020), we have prioritized these studies in females. However, multiple reports from our group established that PAE-related neuropathic pain susceptibility is evident in male rats that follow a comparable timeline of allodynia development and reversal with PAE female rats (Sanchez et al., 2017, 2019). Therefore, we anticipate that dysregulation of circRNAs will be present in male rats, although sex-specific patterns of PAE-induced circRNA dysregulation may occur (Paudel et al., 2020; Papageorgiou et al., 2023). Future studies will examine sex-specific differences in circRNA expression during adulthood under PAE conditions.

## Data availability statement

The datasets presented in this study can be found online: https://data.mendeley.com/datasets/mt4fwkzh77/draft?a=8aa0ba.

## Ethics statement

The animal study was reviewed and approved by Institutional Animal Care and Use Committee (IACUC) of The University of New Mexico Health Sciences Center.

## Author contributions

SN, EM, and NM contributed to the conception and design of the study and edited the manuscript. JES, JJS, and EM collected allodynia behavior data. SN, ANP, JES, JJS, and EM collected tissues. SN, MS, and ANP processed the tissues. NM guided designing circRNA-specific primers and primer slope analysis. ANP, AAP, AKF-O, and SN performed the real-time PCRs and statistical analysis. ANP carried out that IPA analysis. SN and MD assisted IPA analysis. ANP and SN wrote the first draft of the manuscript. SD and DS generated Saccharin control and moderate PAE rat offspring and assisted with manuscript preparation. All authors contributed to the article and approved the submitted version.

## Funding

This work was supported by National Institute of Health (NIH) grants R01 AA025967, R21 AA023051, T32 AA014127, P50 AA022534, R01 AA029694, and R01 AA026583.

## Conflict of interest

NM has a financial interest in Circular Genomics Inc., a company focused on the use of circRNAs as biomarkers for psychiatric disorders.

The remaining authors declare that the research was conducted in the absence of any commercial or financial relationships that could be construed as a potential conflict of interest.

## Publisher’s note

All claims expressed in this article are solely those of the authors and do not necessarily represent those of their affiliated organizations, or those of the publisher, the editors and the reviewers. Any product that may be evaluated in this article, or claim that may be made by its manufacturer, is not guaranteed or endorsed by the publisher.
