# Supplementary figures and images for: Prenatal alcohol exposure dysregulates spinal and circulating immune cell circular RNA expression in adult female rats with chronic sciatic neuropathy
Source: Front Neurosci. 2023 Jun 9;17:1180308. doi: 10.3389/fnins.2023.1180308 (PMC10288115; doi:10.3389/fnins.2023.1180308)

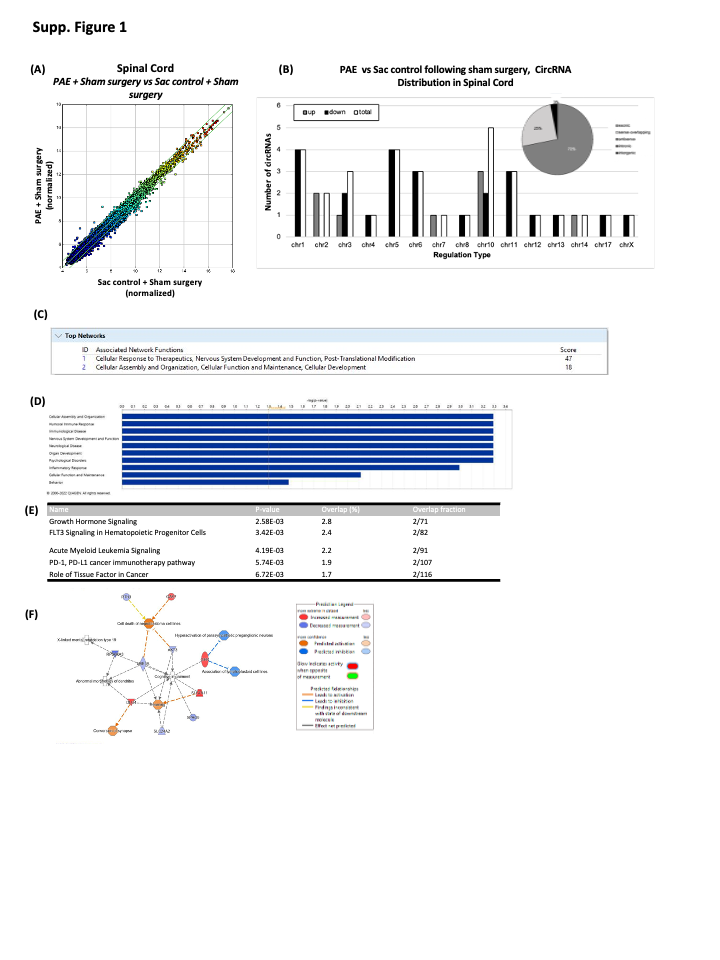

Supplement: Supplementary file 1 [file Image_1.TIFF]

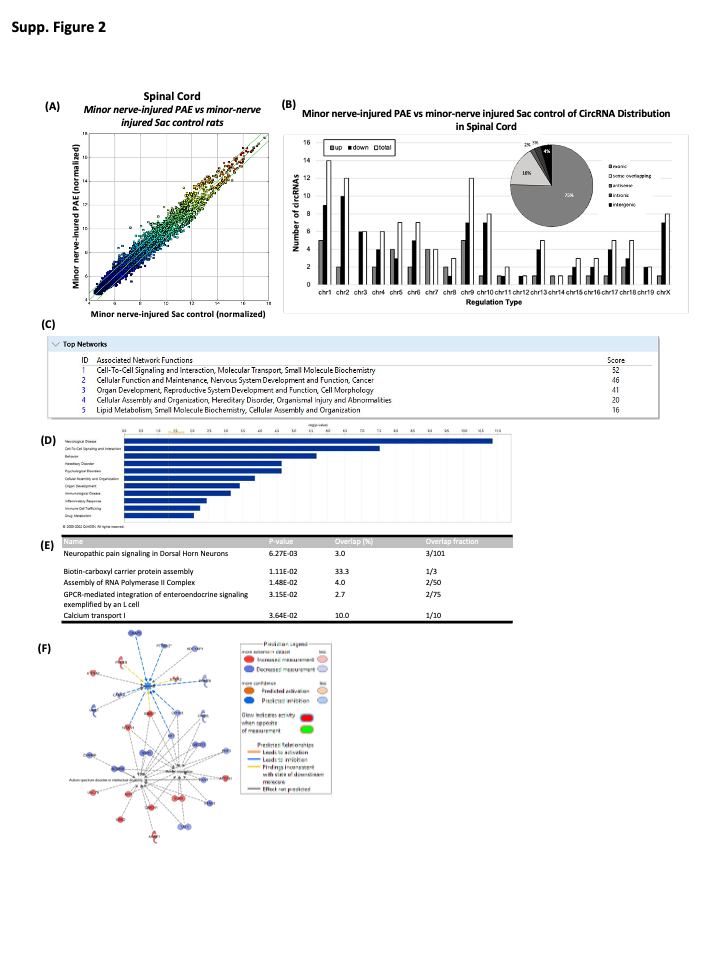

Supplement: Supplementary file 2 [file Image_2.TIFF]

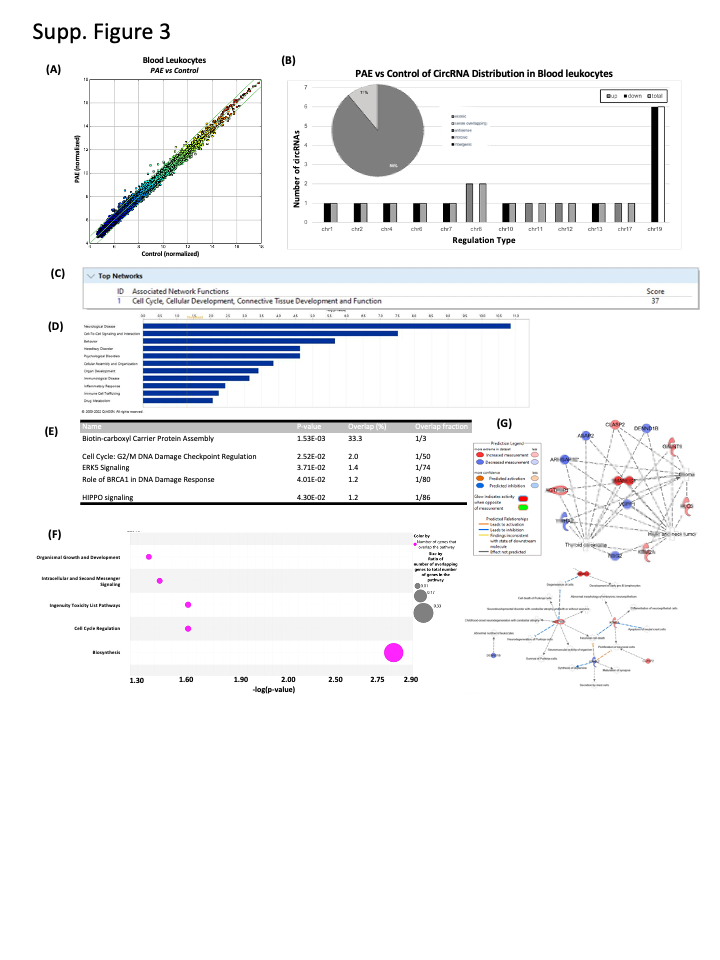

Supplement: Supplementary file 3 [file Image_3.TIFF]

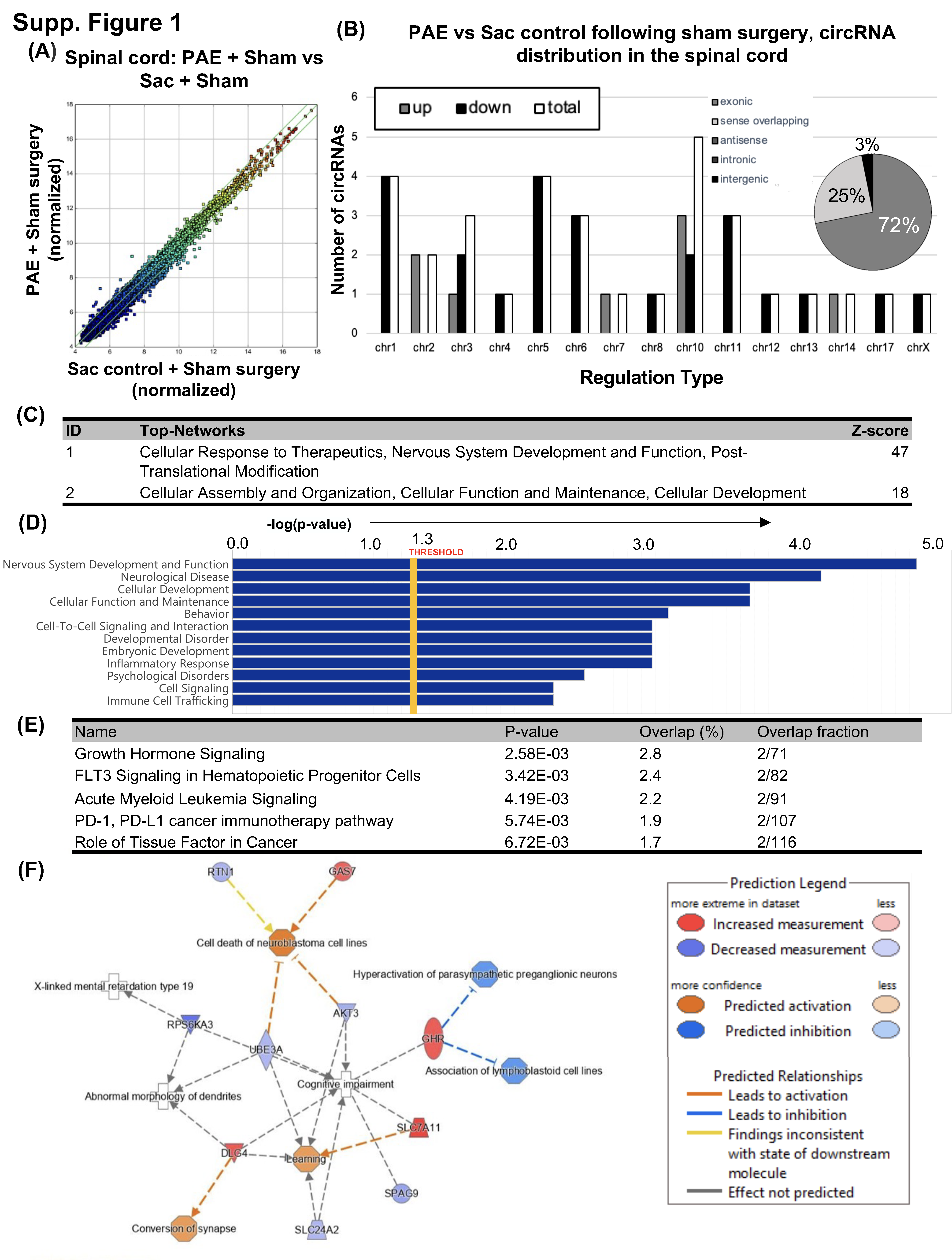

Supplement: Supplementary file 4 [file Image_4.TIFF]

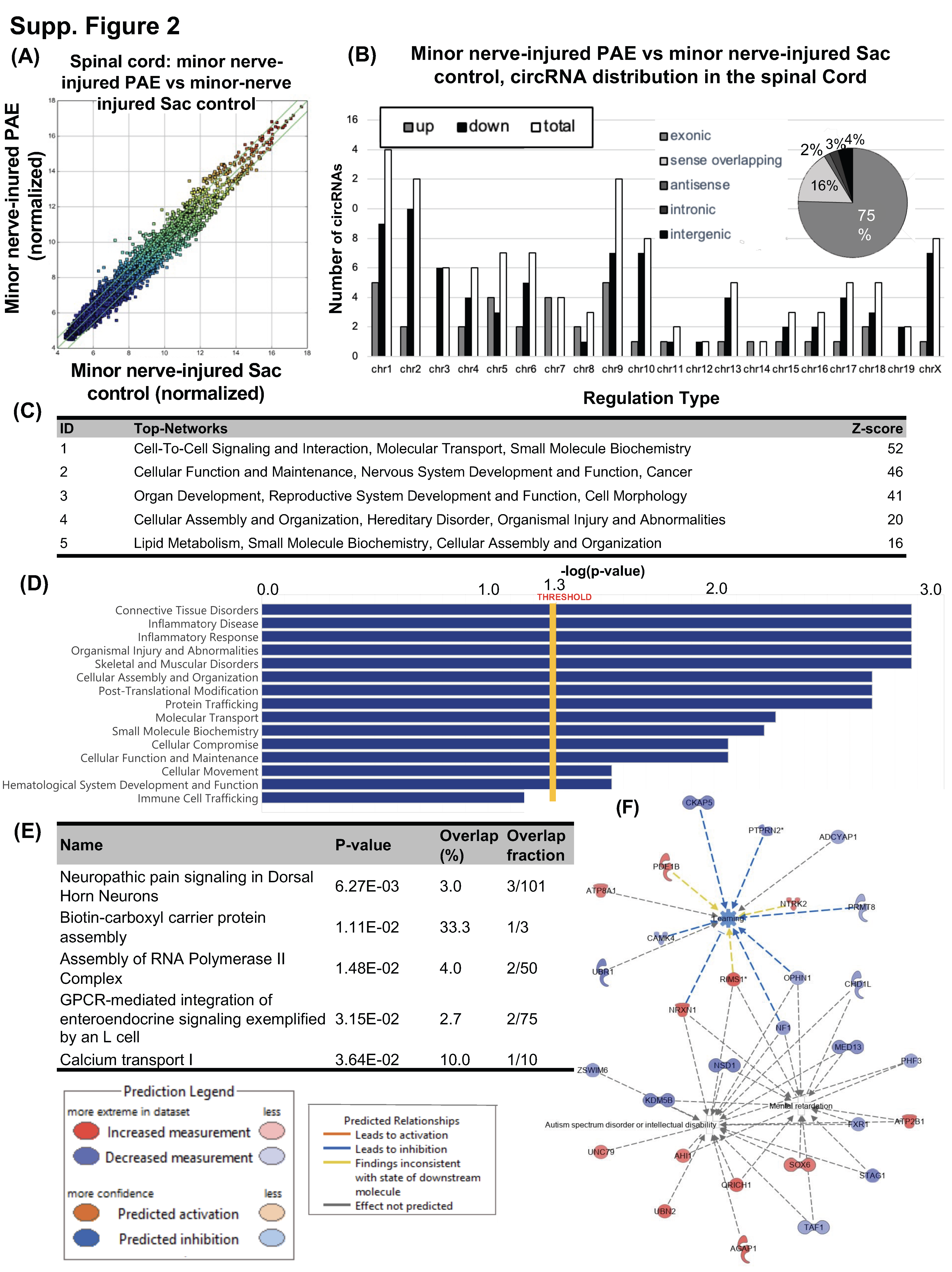

Supplement: Supplementary file 5 [file Image_5.TIFF]

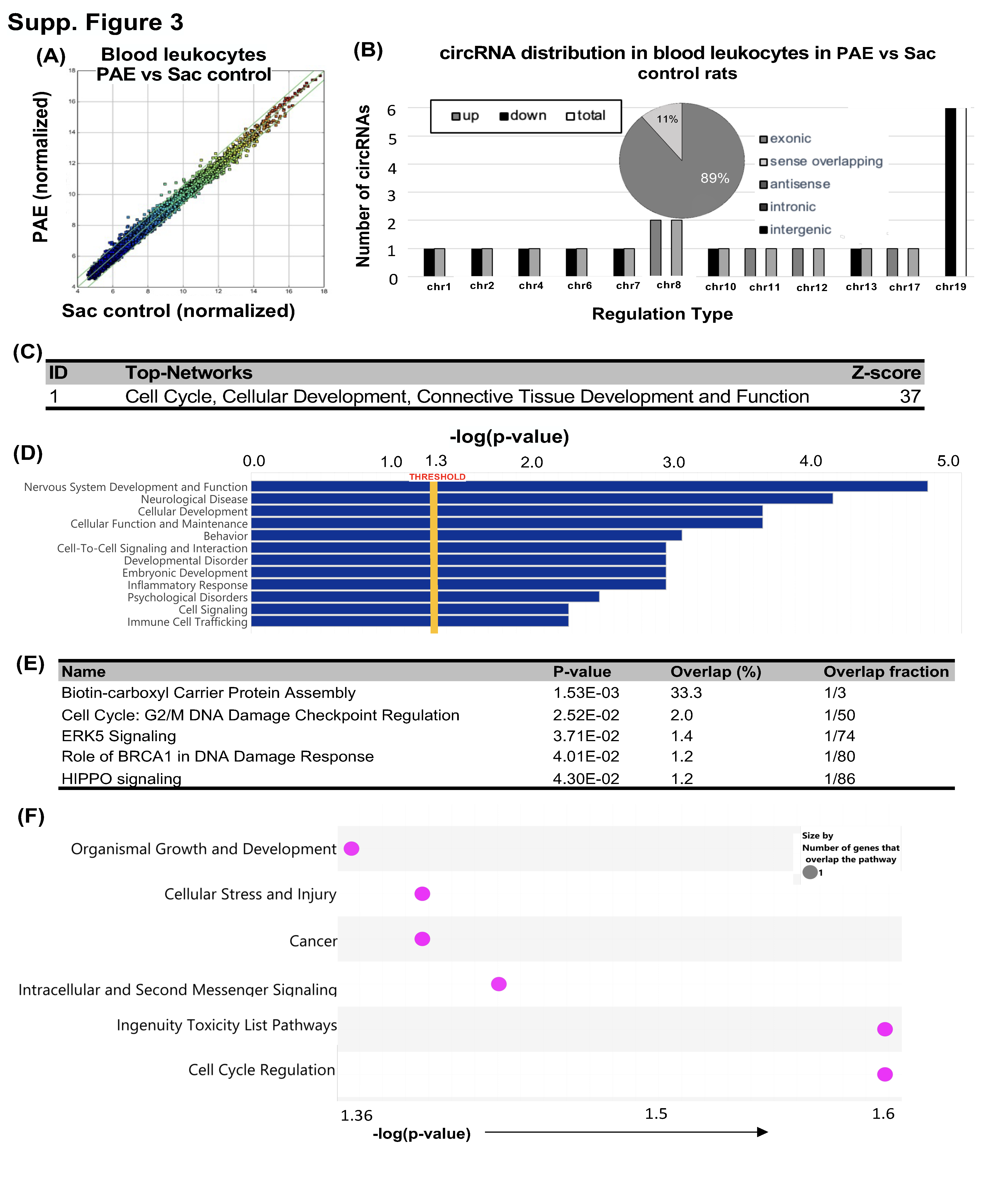

Supplement: Supplementary file 6 [file Image_6.TIFF]
